# Poster Session I - A172 OPTIMIZING THE FECAL IMMUNOCHEMICAL TEST (FIT) POSITIVITY THRESHOLD IN THE NOVA SCOTIA COLON CANCER PREVENTION PROGRAM (CCPP)

**DOI:** 10.1093/jcag/gwaf042.172

**Published:** 2026-02-13

**Authors:** S D Bray, H Petropolis, D Raha, M Stewart

**Affiliations:** Division of Digestive Care & Endoscopy, Dalhousie University, Halifax, NS, Canada; Division of Digestive Care & Endoscopy, Dalhousie University, Halifax, NS, Canada; Nova Scotia Health Authority, Halifax, NS, Canada; Division of Digestive Care & Endoscopy, Dalhousie University, Halifax, NS, Canada

## Abstract

**Background:**

Colorectal cancer (CRC) is the second leading cause of death from cancer in men and third in women in Canada [1]. In 2009, the Nova Scotia (NS) Colon Cancer Prevention Program (CCPP) was the first colon cancer screening program in Canada to provide population-based CRC screening mailing Fecal Immunochemical Testing (FIT) to all residents of NS between ages 50-74 enrolled in the provincial Medical Services Insurance program.

**Aims:**

In 2023, NS Health changed their FIT vendor to a new supplier (*Polmedco OC-Auto Sensor Diana*) and the positivity threshold was decreased from 100 ng/ml to 50 ng/ml, in line with British Columbia. The aim of this study is to evaluate the positive predictive value (PPV) of the new positive threshold compared to the previous. We hypothesize that the PPV at the lower threshold will decrease for detection of all neoplasms.

**Methods:**

We evaluated the PPV of the new FIT threshold in detecting colorectal lesions in the NS population between September 1^st^, 2023 – February 28^th^, 2025. PPV was evaluated for two outcomes: *PPV1* – any pre-cancerous or cancerous lesion (serrated, adenomatous or malignant) and *PPV2* – advanced adenoma or malignancy. *PPV1 & PPV2* were compared across three FIT thresholds: 50 – 74 ng/ml, 75 – 99 ng/ml and 100+ ng/ml using chi-square analysis.

**Results:**

Using the Cancer Care Application of Screening Platform and Event Relationship, 7,271 FIT-positive cases underwent colonoscopy. Males represented 58.9%, with the highest positivity rate in the 70–74 age group (16.1%). *The PPV1* was 87.9% overall, highest in the 70–74 age group (90.3%). *The overall PPV2 was* 32.6%, highest in the 50–54 age group (33.7%). When comparing FIT threshold groups, the 100+ ng/mL group had a significantly higher *PPV1* (88.2%) compared to the 50–74 ng/mL (85.1%) and 75–99 ng/mL (85.5%) groups (p < 0.05), while the lower thresholds did not differ significantly from each other. FIT threshold was significantly associated with *PPV2 (p < 0.001)*, increasing consistently with higher thresholds (Table 1).

**Conclusions:**

Overall, the PPV remains high at 87.9% despite decreasing the qualtified FIT positivity threshold. Interestingly, the *PPV1* did not differ between the two lower threshold groups, raising questions about the optimal cut-off given the added colonoscopy burden at lower positivity thresholds. The significant association between FIT threshold and *PPV2* emphasizes the importance of maintaining a balance between the FIT positive threshold and endoscopy burden. Ongoing economic analyses will assess whether the higher FIT positivity rate at the new threshold provides net benefit through cancer prevention and earlier cancer detection despite increased colonoscopy demand.

1. Canadian Cancer Society. (2024). Colorectal Cancer Statistics. https://cancer.ca/en/cancer-information/cancer-types/colorectal/statistics

A160 Table 1

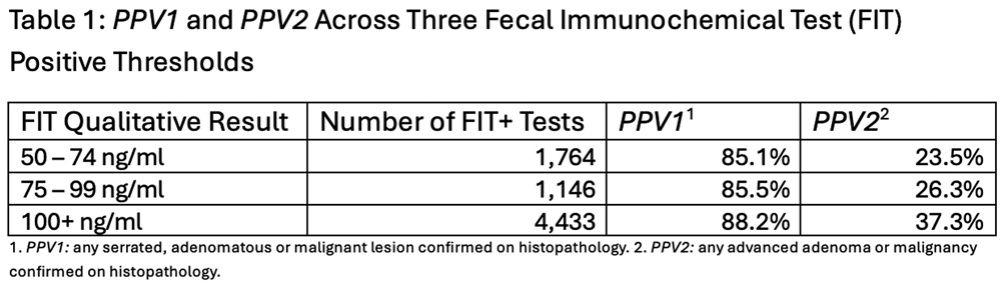

**Funding Agencies:**

None

